# Association between CD4^+^ T cell counts and gut microbiota and serum cytokines levels in HIV-infected immunological non-responders

**DOI:** 10.1186/s12879-021-06491-z

**Published:** 2021-08-03

**Authors:** Danfeng Lu, Jian-Bo Zhang, Yue-Xin Wang, Shi-Tao Geng, Zunyue Zhang, Yu Xu, Shao-You Li, Kun-Hua Wang, Yi-Qun Kuang

**Affiliations:** 1 NHC Key Laboratory of Drug Addiction Medicine, First Affiliated Hospital of Kunming Medical University, Kunming Medical University, 295 Xichang Road, Kunming, Yunnan China; 2 Scientific Research Laboratory Center, First Affiliated Hospital of Kunming Medical University, Kunming, China; 3 Department of Gastrointestinal and Hernia Surgery, First Affiliated Hospital of Kunming Medical University, Kunming, China; 4 Department of Dermatology, Second People’s Hospital of Dali City, Dali, China

**Keywords:** Immunological non-responders, CD4^+^ T cell counts, Gut microbiota, Cytokines, *Ruminococcaceae*

## Abstract

**Background:**

CD4^+^ T cell counts in certain human immunodeficiency virus (HIV)-infected patients called immunological non-responders (INRs) could not return to a normal level even with sustained antiretroviral therapy (ART) because of persistent immune activation, which is associated with pro-inflammatory cytokines production and an altered intestinal microbiome profile. Changes in gut bacterial composition have been linked to low CD4^+^ T cell counts in HIV-infected individuals. However, the association between CD4^+^ T cell counts and gut microbiota community composition and cytokines levels in INRs (CD4^+^ T cell counts < 500 cells/μL) from Yunnan Province, China, has not been previously investigated.

**Methods:**

To address this issue, we carried out a cross-sectional study of 34 HIV-infected INRs. The patients were divided into CD4 count > 200 cells/μL group and CD4 count < 200 cells/μL group. The gut microbiota composition of each subject was analyzed by *16S* rRNA gene sequencing. We also compared CD8^+^ T cell counts, pro-inflammatory cytokines levels, and nutritional status between the two groups.

**Results:**

Compared to INRs with CD4 count > 200 cells/μL, those with CD4 count < 200 cells/μL had a lower CD4/CD8 ratio, lower nutritional status and higher serum levels of tumor necrosis factor (TNF)-α, interferon-γ-inducible protein (IP)-10 and interleukin (IL)-1α. *Ruminococcaceae* was less abundant in the CD4 count < 200 cells/μL group than in the CD4 count > 200 cells/μL group, and difference in alpha diversity was observed between the two groups. Moreover, CD4^+^ T cell counts were negatively associated with TNF-α and IL-1α levels and positively associated with the relative abundance of *Ruminococcaceae*.

**Conclusions:**

Our study demonstrated that lower CD4^+^ T cell counts in INRs are associated with a reduced abundance of *Ruminococcaceae* in the gut and elevated serum pro-inflammatory cytokines levels. Thus, interventions targeting gut microbiota to increase CD4^+^ T cell counts are a potential strategy for promoting immune reconstitution in HIV-infected INRs.

**Supplementary Information:**

The online version contains supplementary material available at 10.1186/s12879-021-06491-z.

## Background

Human immunodeficiency virus (HIV) infection depletes CD4^+^ T cells, leading to the development of acquired immunodeficiency syndrome (AIDS), AIDS-defining opportunistic infections or cancer. CD4^+^ T cell counts circulating in the blood are less than 200 cells/μL in AIDS patients [1, 2]. Although plasma viral load declines to undetectable levels after initiation of antiretroviral therapy (ART), immune reconstitution may not occur; about 10–40% of HIV-infected individuals fail to normalize CD4^+^ T cell counts. These patients, known as immunological non-responders (INRs), have severe immune dysfunction with CD4^+^ T cell counts < 500 or < 350 cells/μL, or in extreme cases, < 200 cells/μL [3, 4].

HIV infection leads to the destruction of the intestinal integrity and mucosa, imbalance of gut microbial community composition, microbial translocation, systemic inflammation, persistent immune activation, pro-inflammatory cytokines release, immune exhaustion, and organ dysfunction [5–11]. The failure of CD4^+^ T cell counts to normalize in INRs may be attributable to immune activation associated with compromised gut barrier immunity and alterations in intestinal microbiome profile [9, 12–15]. It was also reported that ART-treated HIV patients with poor CD4^+^ T cell and CD4/CD8 ratio recovery have increased microbial translocation and elevated levels of pro-inflammatory cytokines [16].

Alpha diversity measures reflect the complexity and/or richness of operational taxonomic units (OTUs). Higher alpha diversity is generally considered as a marker of health; HIV infection status was found to be significantly associated with a decrease in alpha diversity [17]. HIV infection causes a shift in gut microbiota community composition from *Bacteroides* to *Prevotella* predominance [16–19]. Independent of gender and sexual behavior, individuals with HIV infection have a distinct gut microbiome profile characterized by *Gammaproteobacteria* enrichment, *Lachnospiraceae* and *Ruminococcaceae* depletion, and decreased alpha diversity [20]. Compared to ART-naïve HIV progressors, elite controllers were found to harbor a larger number of genera (including a greater abundance of *Succinivibrio*) and had higher richness indices for fecal microbiota [21].

Low CD4^+^ T cell counts (< 200 cells/μL) were shown to be associated with alterations in the bacterial microbiome in an HIV-infected cohort in Uganda [1], and findings from a study of HIV-infected individuals in Beijing found that the abundance of certain bacterial strains was closely related to CD4^+^ T cell numbers regardless of ART [16]. However, there have been no detailed and comprehensive studies on the relationship between immunological status, gut microbiome profile, and inflammation in INRs in Yunnan Province, China. To address this issue, this study investigated the association between CD4^+^ T cell counts (with 200 cells/μL as the cut-off value), gut microbiota community composition and abundance, and serum cytokines levels in HIV-infected INRs in Yunnan.

## Methods

### Study design

A total of 34 HIV-infected INRs (CD4 count < 500 cells/μL), receiving sustained ART (Table 1) with undetectable (< 50 copies/mL) plasma HIV-1 RNA level for at least 24 months were recruited at the Second People’s Hospital of Dali City in Dali, Yunnan, China from June 2019 to November 2019. The patients were divided into two groups according to CD4^+^ T cell counts (CD4 count > 200 cells/μL (n = 17) and CD4 count < 200 cells/μL (n = 17) after more than two years of ART, respectively). All subjects were ≥ 18 years old and provided written informed consent before participating in the study, which was approved by the Human Clinical Research Ethics Committee of the First Affiliated Hospital of Kunming Medical University (No. 2018L43). All experiments were performed in accordance with the approved guidelines and regulations according to the principles expressed in the Declaration of Helsinki, and the experimental protocols were approved by the institutional review board of Kunming Medical University. The clinical characteristics of the study participants are shown in Table 1.

**Table 1:** Study cohort characteristics

	CD4 < 200 (N = 17)	CD4 > 200 (N = 17)	*P* value
Mean age (years)	50 ± 9	47 ± 7	0.2619
Weight (kg)	54.71 ± 9.71	58.18 ± 8.63	0.1895
BMI (kg/m^2^)	21.19 ± 3.52	21.69 ± 2.92	0.4691
Time on ART (years)	7 ± 3	9 ± 2	0.0951
Gender, N (%)			
Male	7 (41.2)	8 (47.1)	> 0.9999
Female	10 (50.0)	9 (52.9)	
Ethnicity, N (%)			
Han	4 (23.5)	4 (23.5)	> 0.9999
Other minorities	13 (58.8)	13 (76.5)	
Acquisition of HIV, N (%)			
Injection drug use	3 (17.6)	1 (5.9)	0.7309
Sexual route	12 (70.6)	14 (82.3)	
Unknown	2 (11.8)	2 (11.8)	
T cells (cells/μL)			
CD4^+^ T cell counts before ART	159 ± 143	210 ± 149	0.1425
CD3^+^ T cell counts	642 ± 263	764 ± 178	0.0518
CD4^+^ T cell counts	149 ± 38	266 ± 57	< 0.0001*
CD8^+^ T cell counts	448 ± 233	454 ± 171	0.4902
CD4/CD8 ratio	0.40 ± 0.20	0.72 ± 0.45	0.0033*
Nadir CD4^+^ T cell counts	144 ± 37	239 ± 36	< 0.0001*
ART regimens, N (%)			
NRTI + NNRTI	13 (76.5)	17 (100)	0.1026
NRTI + PI	4 (23.5)	0 (0)	
3TC + AZT + EFV	4 (23.5)	5 (29.4)	0.1404
3TC + AZT + NVP	3 (17.6)	6 (35.3)	
3TC + TDF + EFV	5 (29.4)	5 (29.4)	
3TC + AZT + LPV	2 (11.8)	0 (0)	
3TC + TDF + NVP	1 (5.9)	1 (5.9)	
TDF + AZT + LPV	1 (5.9)	0 (0)	
3TC + LPV	1 (5.9)	0 (0)	

Data are presented as Mean ± SD unless otherwise indicated. *P* values were calculated using Mann–Whitney test. Red marked lines represent *P* < 0.05. *BMI* body mass index, *HIV* human immunodeficiency virus, *ART* antiretroviral therapy, *NRTI* nucleoside reverse transcriptase inhibitor, *NNRTI* non-nucleoside reverse transcriptase inhibitor, *PI* protease inhibitor, *3TC* lamivudine, *AZT* zidovudine, *EFV* efavurebz, *NVP* nevirapine, *TDF* tenofovir, *LPV* lopinavir

*P < 0.05

Subjects who had used antibiotics, probiotics, or prebiotics or had experienced diarrhea or digestive symptoms within the previous 3 months were excluded, as were those with active infection or co-infection with hepatitis B virus (HBV) and HCV.

### Clinical data

Fasting blood was collected and the serum was immediately separated by centrifugation and stored at − 80°C until analysis. The samples were used for measurement of systemic inflammation markers and laboratory tests including blood routine and blood biochemical tests.

A heparin anticoagulation vacuum tube was used to collect about 5 mL of venous blood. The absolute numbers of CD3^+^, CD4^+^, and CD8^+^ T lymphocytes were determined using the BD Multitest IMK Kit with BD Trucount tubes on a BD FACSCalibur flow cytometer (BD Biosciences, Franklin Lakes, NJ, USA).

### Assessment of nutritional status

Nutritional status was determined by the multiple-frequency bioelectrical impedance method using a body composition analyzer (InBody S10; Biospace, Seoul, Korea). The parameters, which included segmental water, segmental lean, and body mass index (BMI), were recorded immediately after measurement.

### Multiplex assay

The levels of ten pro-inflammatory cytokines including interleukin (IL)-1β, IL-6, IL-1α, tumor necrosis factor (TNF)-α, TNF-β, interferon-gamma-inducible protein (IP)-10, monocyte chemotactic protein (MCP)-1, soluble cluster of differentiation 14 (sCD14), D-dimer and C-reactive protein (CRP) were quantified with the FlexMAP 3D with MILLIPLEX Analyst (Millipore, Billerica, MA, USA) and the Human Premixed Multi-Analyte Kit (R&D Systems) according to the manufacturer’s instructions.

### DNA extraction and sequencing

Fecal samples were sampled in sterile containers by the patients and immediately stored at – 80 °C until processing. Bacterial DNA was extracted using the QIAamp PowerFecal DNA kit (Qiagen, Valencia, CA, USA) according to the manufacturer’s instructions. The concentration of DNA extracted from the samples was measured with a NanoDrop spectrophotometer (Thermo Fisher Scientific, Waltham, MA, USA), and was adjusted to 12 ng/μL for experiments. Extracted genomic DNA was PCR-amplified with degenerate PCR primers (515F, 5′-GTCCAGCMGCCGCGGTAA-3′; 806R, 5′-GGACTACHVGGGTWTCTAAT-3′) targeting the V4 region of the bacterial *16S* rRNA gene. Both forward and reverse primers were tagged with adapter, pad, and linker sequences. PCR enrichment was performed using equal amounts (30 ng) of DNA from each sample in a 50 μL reaction. PCR products were purified using the Agencourt AMPure XP beads (Beckman Coulter, Brea, CA, USA) and recovered in the elution buffer. Libraries were quantified using Agilent 2100 Bioanalyzer and sequencing was performed at the Beijing Genomics Institute (BGI, Wuhan, China) on a HiSeq 2500 platform (Illumina, San Diego, CA, USA) according to the manufacturer’s standard protocol, generating 250-bp paired-end reads. Raw sequence data generated for this study are available in the Sequence Read Archive under BioProject accession PRJNA695425.

### Analysis of sequencing data

Raw reads were filtered to cut off adaptors and low-quality and ambiguous bases, and paired-end reads were merged using the Fast Length Adjustment of Short reads v1.2.11 program (https://ccb.jhu.edu/software/FLASH/) and filtered under specific conditions to obtain high-quality clean tags with FQTRIM v0.94 (https://ccb.jhu.edu/software/fqtrim/). Chimeric sequences were compared with those in the Gold database using UCHIME v4.2.40 (https://drive5.com/usearch/manual/uchime_algo.html). After dereplication using DADA2 (https://benjjneb.github.io/dada2/index.html), we obtained the feature table and feature sequence. Representative OTU sequences were taxonomically assigned using the GreenGenes database v201305 (https://greengenes.secondgenome.com/). Alpha and beta diversity were calculated using QIIME2 (https://qiime2.org/). Using the SILVA (release 132) classifier (https://www.arb-silva.de/), feature abundance was normalized using the relative abundance of each sample. Alpha diversity was used to analyze the complexity of species diversity in each sample based on five indices including Chao1, Observed species, Goods coverage, Shannon, and Simpson. BLAST (https://blast.ncbi.nlm.nih.gov/Blast.cgi) was used for sequence alignment, and the feature sequences were annotated with the SILVA database for each representative sequence. Diagrams were generated using R package v3.5.2 (https://www.r-project.org/).

### Statistical analysis

Alpha diversity measures (Shannon and Simpson diversity indices) were calculated using R package V3.5.2 and compared with the Wilcoxon matched-pairs signed-rank test. Beta diversity analyses based on unweighted UniFrac distance matrices were performed, and differences in microbial community composition between groups were evaluated by Analysis of Similarities (ANOSIM). Principal coordinates analysis (PCoA) or nonmetric multidimensional scaling (NMDS) was used as an ordination method to visualize unweighted UniFrac dissimilarity values in the two groups. Relative taxa abundance (at the family level) and various parameters were compared between the two groups with the Mann–Whitney test. Data are reported as mean ± standard deviations (SD) and were analyzed using Prism 8.0 software (GraphPad, La Jolla, CA, USA). Statistical significance was defined as *P* < 0.05.

## Results

### Characterization of the study cohort

A total of 34 HIV-infected participants, aged 33–69 years, were enrolled in this study. No differences were observed between groups in terms of mean age, BMI, and time on ART (Table 1). In the CD4 count > 200 cells/μL group, 8 patients (47.1%) were male and 4 (23.5%) were of Han ethnicity; in the CD4 count < 200 cells/μL group, 7 (41.2%) were male and 4 (23.5%) were of Han ethnicity. In terms of HIV transmission routes, 3 (17.6%) and 12 (70.6%) individuals in the CD4 count < 200 cells/μL group were infected through intravenous drug use and sexual contact, respectively; in the CD4 count > 200 cells/μL group, the numbers were 1 (5.9%) and 14 (82.3%), respectively. For two patients (11.8%) in each group, the route of transmission was unknown. The demographic characteristics of the cohort are shown in Table 1.

Patients with CD4 count < 200 cells/μL had lower CD4^+^ T cell counts (*P* < 0.0001) nadir CD4^+^ T cell counts (*P* < 0.0001), and CD4/CD8 ratio (*P* = 0.0033) than those in the CD4 count > 200 cells/μL group. Additionally, the numbers of CD3^+^ T cells (*P* = 0.0518) and CD8^+^ T cells (*P* = 0.4902) were lower in the CD4 count < 200 cells/μL group, but not significant. CD4^+^ T cell counts were higher in the CD4 count > 200 cells/μL group than those in the CD4 count < 200 cells/μL group before the initiation of ART, although the difference between the two groups was nonsignificant (*P* = 0.1425).

### Serum levels of pro-inflammatory cytokines are elevated in INRs with low CD4^+^ T cell counts

Chronic immune activation and inflammation persist in HIV-infected patients even when ART effectively inhibits viral replication. This was confirmed by our observation that serum levels of the pro-inflammatory cytokines TNF-α (*P* = 0.0070), IL-1α (*P* = 0.0141) and IP-10 (*P* = 0.0261) were higher in the CD4 count < 200 cells/μL group than in the CD4 count > 200 cells/μL group (Table 2). However, the latter patients had a higher CRP level, although this was not a statistically significant difference (*P* = 0.0834).

**Table 2:** Serum levels of pro-inflammatory cytokines are elevated in INRs with low CD4^+^ T cell counts

Cytokines (pg/mL)	CD4 < 200 (N = 17)	CD4 > 200 (N = 17)	*P* value
TNF-α	11.14 ± 1.83	9.62 ± 1.65	0.0070*
IP-10	87.54 ± 51.21	53.61 ± 31.60	0.0261*
IL-1α	39.80 ± 6.56	33.91 ± 7.26	0.0141*
MCP-1	163.20 ± 45.38	136.90 ± 42.65	0.1309
D-dimer	1337.00 ± 864.50	1218.00 ± 984.60	0.1705
sCD14	1,737,315 ± 463,779	1,617,557 ± 532,146	0.2208
TNF-β	2.02 ± 0.93	1.76 ± 0.65	0.4279
IL-1β	8.55 ± 1.93	7.94 ± 1.34	0.2313
IL-6	40,486 ± 6550	36,234 ± 7964	0.2451
CRP	2,147,413 ± 2,167,463	4,394,271 ± 3,818,065	0.0834

Data are presented as Mean ± SD unless otherwise indicated. *P* values were calculated using Mann–Whitney test. Red marked lines represent *P* < 0.05. *TNF-α* tumor necrosis factor-alpha, *IP-10* interferon gamma-inducible protein 10, *IL-1α* interleukin 1 alpha, *MCP-1* monocyte chemotactic protein-1, *sCD14* soluble cluster of differentiation 14, *CRP* C reactive protein

*P < 0.05

### INRs with low CD4^+^ T cell counts have lower nutritional status

The results of the body composition analysis indicated that the levels of segmental water (left arm [LA]) (*P* = 0.0419), segmental water (trunk [TR]) (*P* = 0.0437), segmental lean (right arm [RA]) (*P* = 0.0496), segmental lean (LA) (*P* = 0.0496), and segmental lean (TR) (*P* = 0.0437) were lower in the CD4 count < 200 cells/μL group than in the CD4 > 200 cells/μL group (Table 3), suggesting that INRs with fewer CD4^+^ T cells are at higher risk for loss of muscle and edema. In terms of blood parameters, hemoglobin level (*P* = 0.0172) and hematocrit (HCT) (*P* = 0.0493) were markedly lower in INRs with CD4 count < 200 cells/μL (Table 3). The above results suggest that INRs in the CD4 count < 200 cells/μL group have a lower nutritional status.

**Table 3:** INRs with low CD4^+^ T cell counts have lower nutritional status

	CD4 < 200 (N = 17)	CD4 > 200 (N = 17)	*P* value
Segmental water analysis (L)			
Segmental Water (RA)	1.56 ± 0.32	2.04 ± 1.03	0.0518
Segmental Water (LA)	1.52 ± 0.32	2.25 ± 1.98	0.0419*
Segmental Water (TR)	14.15 ± 1.97	17.43 ± 7.64	0.0437*
Segmental Water (RL)	6.11 ± 1.01	6.85 ± 1.23	0.0716
Segmental Water (LL)	6.04 ± 0.94	6.70 ± 1.17	0.1005
Segmental lean analysis (kg)			
Segmental Lean (RA)	2.02 ± 0.41	2.65 ± 1.39	0.0496*
Segmental Lean (LA)	1.96 ± 0.41	2.94 ± 2.71	0.0496*
Segmental Lean (TR)	18.25 ± 2.57	22.51 ± 9.89	0.0437*
Segmental Lean (RL)	7.88 ± 1.31	8.84 ± 1.61	0.0744
Segmental Lean (LL)	7.79 ± 1.22	8.63 ± 1.51	0.1004
Blood routine			
Hemoglobin (g/L)	139.20 ± 14.35	150.10 ± 13.77	0.0172*
HCT (%)	0.40 ± 0.039	0.43 ± 0.043	0.0493*

Nutritional status in the CD4 count > 200 group and the CD4 count < 200 group was assessed. Data are presented as Mean ± SD unless otherwise indicated. *P* values were calculated using Mann–Whitney test. Red marked lines represent *P* < 0.05. *RA* right arm, *LA* left arm, *TR* trunk, *RL* right leg, *LL* left leg, *HCT* hematocrit

*P < 0.05

### Gut microbial diversity is altered with reduced CD4^+^ T cell counts

**Fig. 1:**
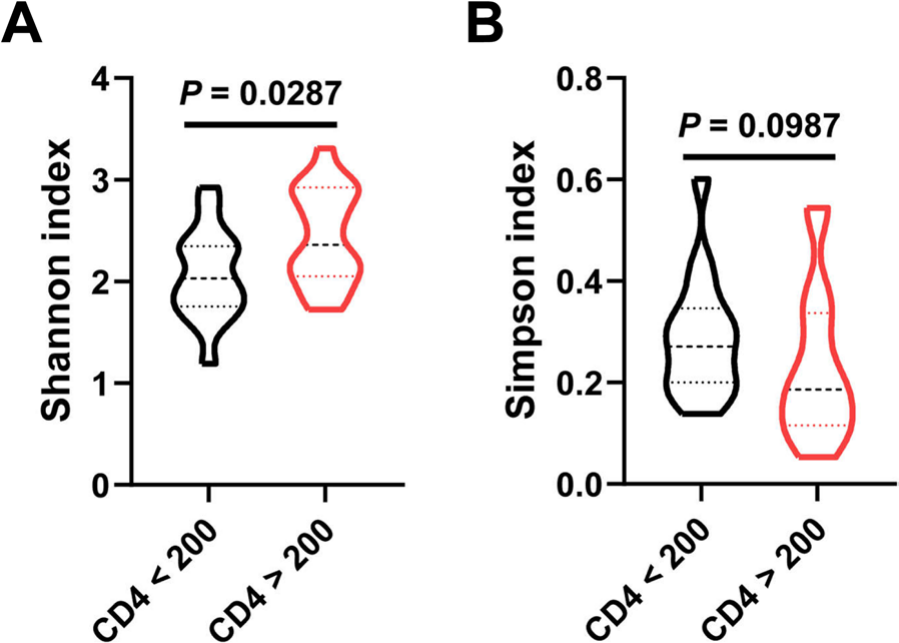
Alpha diversity of gut microbiota in INRs. **A**, **B** Alpha diversity is predicted by the Shannon (**A**) and Simpson (**B**) indexes. *P* values are from the Wilcoxon matched-pairs signed-rank test comparing diversity metrics between the two groups

We compared the alpha diversity of intestinal microbiota between the two groups to determine whether differences in CD4 levels affected the stability of gut microbial communities. The abundance of OTUs differed between groups based on the Shannon index (*P* = 0.0287; Fig. 1A), although the Simpson index did not show a significant difference (*P* = 0.0987; Fig. 1B).

To determine how CD4^+^ T cell counts could affect microbial diversity in INRs, we examined beta diversity across samples. By PCoA and NMDS analysis based on unweighted UniFrac distances, we found that bacterial community structure differed to a certain extent between the two groups; ANOSIM analysis of the distance matrices showed that these differences were not statistically significant (PCoA: *P* = 0.111; Additional file 1: Fig. S1A; NMDS: *P* = 0.101, Additional file 1: Fig. S1B).

### ***Ruminococcaceae*** abundance in the gut is reduced in INRs with low CD4^+^ T cell counts

**Fig. 2:**
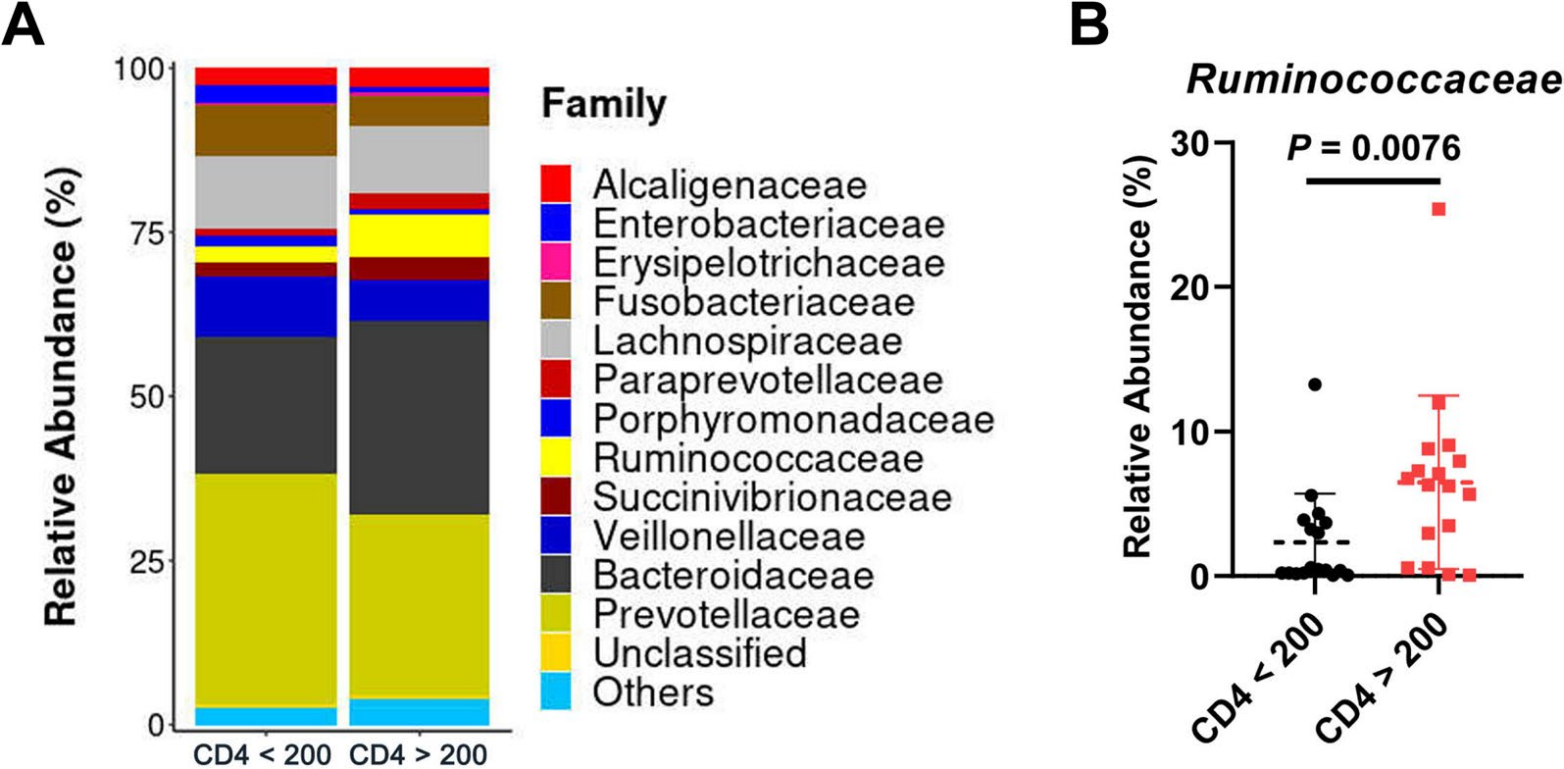
*Ruminococcaceae* abundance in INRs. **A** Relative distributions of families in the two groups are shown. Each bar represents the mean relative abundance of the microbial family. **B** *Ruminococcaceae* abundance was lower (*P* = 0.0076) in the CD4 count < 200 cells/µL group compared to the CD4 count > 200 cells/µL group. *P < 0.05 (Mann–Whitney test)

To determine whether these changes in gut microbial community composition are associated with CD4^+^ T cell counts, we compared the abundance of bacterial taxa between the CD4 count > 200 cells/μL group and the CD4 count < 200 cells/μL group. The abundance of *Ruminococcaceae*, *Succinivibrionaceae*, and *Bacteroidaceae* were decreased whereas that of *Enterobacteriaceae*, *Fusobacteriaceae*, *Veillonellaceae* and *Prevotellaceae* were increased in the CD4 count < 200 cells/μL group compared to the CD4 count > 200 cells/μL group (Fig. 2A); of these, only *Ruminococcaceae* showed significantly different enrichment between the two groups (*P* = 0.0076; Fig. 2B), although the abundance of *Bacteroidaceae* was slightly higher (*P* = 0.3223; Additional file 1: Fig. S2A) and that of *Prevotellaceae* (*P* = 0.7339; Additional file 1: Fig. S2B) along with the *Prevotellaceae*/*Bacteroidaceae* ratio (*P* = 0.4023; Additional file 1: Fig. S2C) were lower in the CD4 > 200 group.

### Pro-inflammatory cytokines levels are correlated with clinical parameters in INRs

**Fig. 3:**
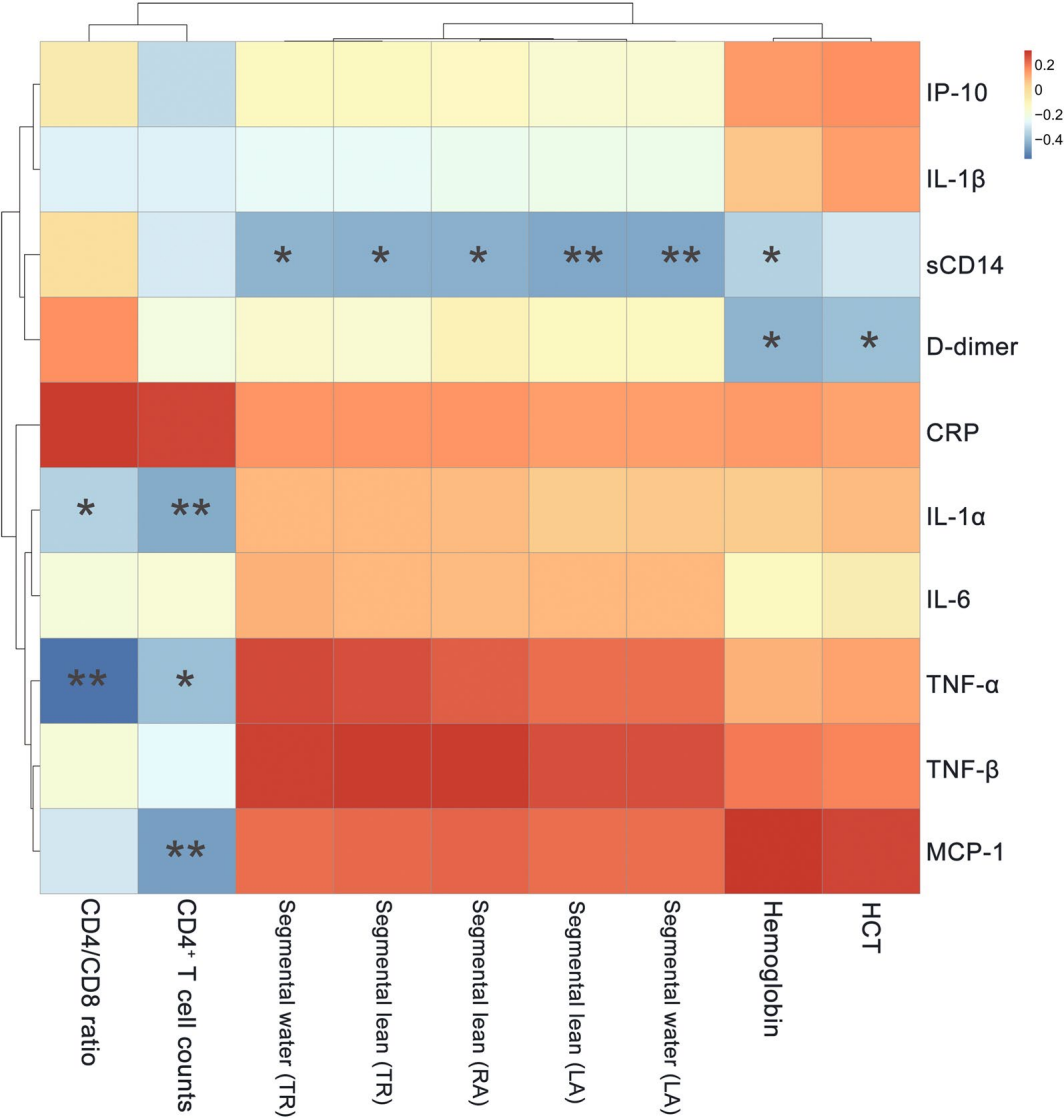
Correlation between clinical variables and pro-inflammatory cytokine levels. Red and blue shading indicate positive and negative associations, respectively. *P < 0.05; **P < 0.01 (Pearson’s correlation)

We analyzed the relationship between clinical parameters and pro-inflammatory cytokines by constructing a heatmap to identify significant correlations (r > 0.3 or < − 0.3) between these variables (Fig. 3). Serum levels of TNF-α and IL-1α were negatively correlated with CD4^+^ T cell counts (r = − 0.400 to − 0.440, *P* < 0.05) and CD4/CD8 ratio (r = − 0.350 to 0.560, *P* < 0.05); MCP-1 level was negatively correlated with CD4^+^ T cell counts (r = − 0.470, *P* < 0.01); and sCD14 level was negatively associated with hemoglobin level as well as segmental water (LA), segmental water (TR), segmental lean (LA), segmental lean (RA) and segmental lean (TR) (r = − 0.300 to − 0.440, *P* < 0.05). D-dimer level was also negatively correlated with HCT and Hemoglobin level (r = − 0.400 to − 0.420, *P* < 0.05).

### Abundance of specific families of gut bacteria is correlated with clinical variables in INRs

**Fig. 4:**
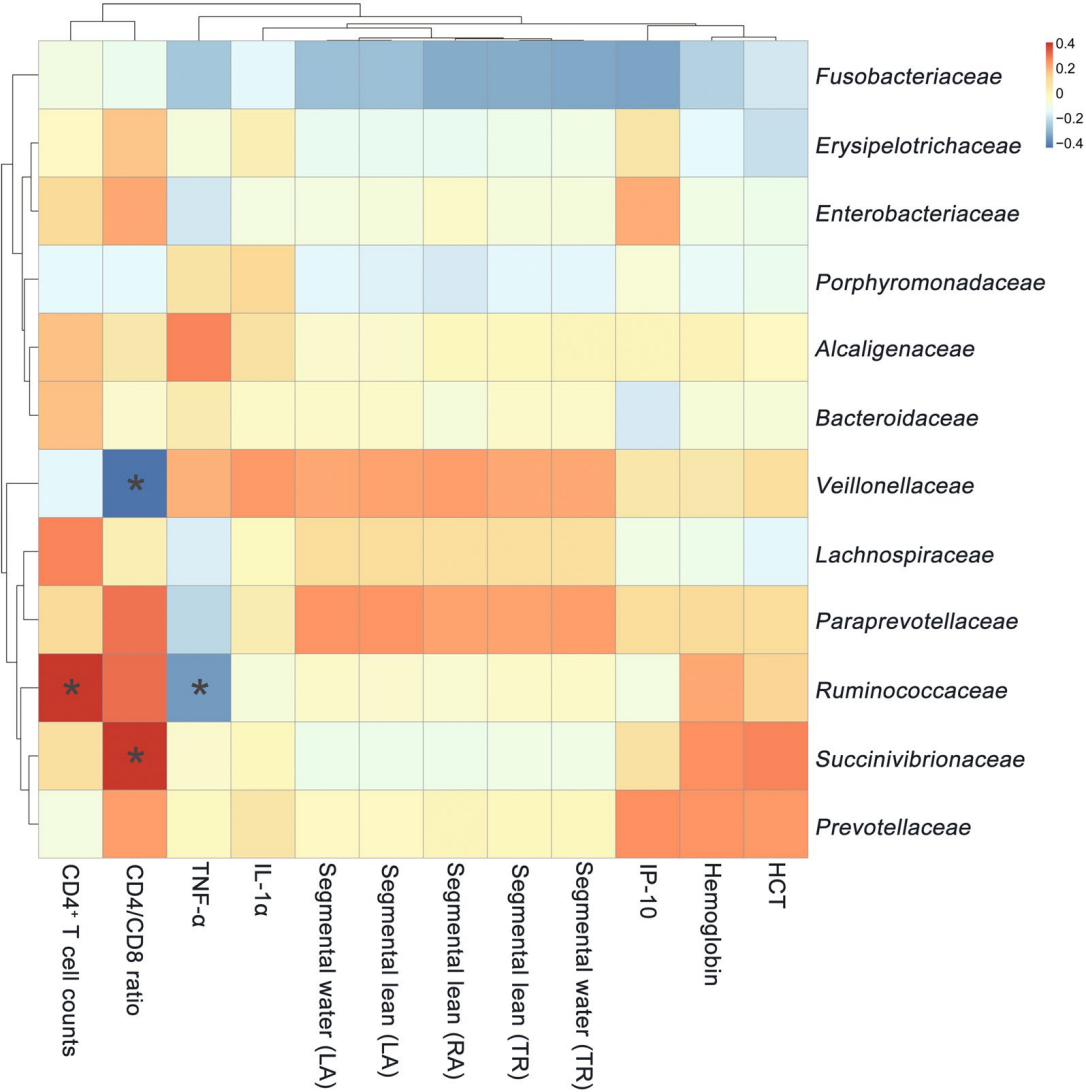
Correlation between clinical variables and the abundance of specific families of gut microbiota. Red and blue shading indicate positive and negative associations, respectively. *P < 0.05; **P < 0.01 (Pearson’s correlation)

To determine the relationship between clinical variables and gut microbiota community composition and diversity at the family level, we constructed a heatmap and analyzed the correlations between variables (r > 0.3 or < − 0.3) (Fig. 4). CD4/CD8 ratio was negatively correlated with the relative abundance of *Veillonellaceae* (r = − 0.440, *P* < 0.05) and had a positive relationship with that of *Succinivibrionaceae* (r = 0.410, *P* < 0.05). The relative abundance of *Ruminococcaceae* was positively associated with CD4^+^ T cell counts (r = 0.400, *P* < 0.05) and negatively correlated with serum level of TNF-α (r = − 0.360, *P* < 0.05).

## Discussion

The results of the present study demonstrated that INRs with lower CD4^+^ T cell numbers have increased inflammation as well as the lower relative abundance of the beneficial bacteria and reduced diversity of bacteria in the gut. Moreover, CD4^+^ T cell counts were closely associated with serum pro-inflammatory cytokines levels and the abundance of *Ruminococcaceae* in the gut.

The homeostasis of the immune system in HIV-infected individuals is partly affected by changes in cytokines levels. Dysregulation of both pro-inflammatory and anti-inflammatory cytokines production contributes to the immune impairment that is associated with the progression of HIV infection to AIDS [22]. Compared to patients with CD4 count > 200 cells/μL, serum levels of pro-inflammatory cytokines including TNF-α, IP-10 and IL-1α were increased whereas CRP level was only slightly decreased in patients with CD4 count < 200 cells/μL. These results suggest that chronic inflammation is exacerbated in HIV-infected INRs with fewer CD4^+^ T cells.

Malnutrition, as reflected by reduced levels of hemoglobin and vitamin D, was found to be associated with elevated levels of sCD14 and D-dimer in HIV/AIDS patients with immune reconstitution inflammatory syndrome [23]. Malnutrition skews immune cells toward an inflammatory state [24, 25]. We found here that parameters related to nutritional status were negatively correlated with sCD14 and D-dimer levels, which implies nutritional intervention is a potential strategy for immune reconstitution in INRs. Elevated plasma levels of inflammatory cytokines perturb T cell homeostasis in HIV-infected patients, resulting in the depletion of CD4^+^ T cells and expansion of CD8^+^ T cell pool [22, 26]. Accordingly, in the present study, there was a negative correlation between serum levels of pro-inflammatory cytokines such as TNF-α and IL-1α and CD4^+^ T cell counts or CD4/CD8 ratio. Our finding that MCP-1 level was negatively correlated with CD4^+^ T cell counts is consistent with a previous report [27].

The gut microbiome of HIV-infected individuals is characterized by enrichment of pro-inflammatory bacterial taxa such as *Erysipelotrichaceae*, *Enterobacteriaceae*, *Desulfovibrionaceae*, and *Fusobacteria* and depletion of anti-inflammatory taxa such as *Lachnospiraceae*, *Ruminococceae*, *Bacteroides*, and *Rikenellaceae* [6, 17, 20, 28, 29]. *Ruminococcaceae* family members are the main producers of short-chain fatty acids, which induce the differentiation of anti-inflammatory regulatory T cells [20, 29]. Our correlation analysis indicated that there was a negative correlation between *Ruminococcaceae* abundance and TNF-α level. The observed positive correlation between *Ruminococcaceae* abundance and CD4^+^ T cell counts is consistent with the previous findings that a higher abundance of the bacterial genera *Faecalibacterium*, *Ruminococcus,* and *Akkermansia* was associated with increased white blood cell counts and *Ruminococcus* enrichment was positively correlated with lymphocyte rates [30]. The study conducted in Uganda reported an altered abundance of *Veillonellaceae* in HIV-infected patients with CD4 count < 200 cells/μL and enrichment of *Succinivibrionaceae* in those with CD4 count > 200 cells/μL [1]. Members of the *Succinivibrionaceae* family with anti-inflammatory activity are associated with ART-related immune recovery, and the genus *Succinivibrio* was found to be more highly represented in elite controllers [21]. Conversely, the abundance of *Veillonellaceae* was increased in chronically ART-naïve individuals compared to those with HIV seronegative status [19]. Moreover, *Veillonella* abundance was correlated with elevated levels of pro-inflammatory cytokines [28]. This could explain our finding that CD4/CD8 ratio was negatively correlated with the relative abundance of *Veillonellaceae* and positively associated with that of *Succinivibrionaceae*. Thus, probiotics/prebiotics or fecal microbiota transplantation could potentially be used to restore intestinal homeostasis and promote immune reconstitution in HIV-infected INRs [6, 10].

Our study has some limitations. Firstly, it was a cross-sectional study based on a small number of Chinese subjects and there was no reliable and detailed information on AIDS events. Secondly, we did not recruit healthy control individuals with normal CD4^+^ T cell counts and immunological responders (IRs) with CD4^+^ T cell counts > 500 cells/μL for comparison. Thirdly, we did not exclude the effects of HIV transmission routes on gut microbiota community composition. The use of methamphetamine and cannabis was found to affect the gut microbiota profile of HIV/AIDS patients [28, 31, 32]. In addition, drug use increased HIV infection and viral replication in immune cells, which accelerated the progression of AIDS [33]. However, the patients in this study were successfully rehabilitated from drug abuse. Fourthly, the patient exclusion criteria did not include gender and sexual behavior; however, alterations in gut microbiota have been reported in HIV-infected patients regardless of sex and sexual activities [20]. Fifthly, Mutlu et al. found that the microbiota of mucosal samples in HIV-positive subjects has more profound changes [34]. However, considering the ethical issues and the difficulties in obtaining gut mucosal biopsies, we did not use gut mucosal specimens to study the microbiome. Finally, we did not examine differences in T cells phenotype/function between the CD4 count > 200 cells/μL group and the CD4 count < 200 cells/μL group because these data were unavailable. In the future, we intend to explore the microbiome of INRs from a functional standpoint, as restoring the function of gastrointestinal microbiota is a potential therapeutic strategy [10]. We will also measure metabolite levels in gut bacteria and plasma samples to identify potential biomarkers related to immune recovery [35, 36].

Increased α-diversity is generally considered to be a marker of health and while decreased diversity has been associated with a variety of disease states [32]. Our results show that if the CD4^+^ T cell counts of INRs are > 200 cells/μL, they are healthier. Although the previous study found that nucleoside reverse transcriptase inhibitors (NRTIs) and protease inhibitors (PIs) regimen does reduce α-diversity [37], we excluded four patients receiving the combination of NRTIs and PIs in the patients with CD4 count < 200 cells/μL and found that difference in α-diversity was still observed between the two groups (unpublished data). In addidtion, the immune situation of INRs with CD4 count < 200 cells/μL may be worse than that of INRs with CD4 count > 200 cells/μL, but the previous study revealed that immunological responders (IRs) with CD4 count > 350 cells/μL had a higher CRP level yet not statistically significant than INRs with CD4 count < 350 cells/μL before the prebiotic intervention. More seriously, INRs with CD4 count < 350 cells/μL had a significantly higher CRP level after the prebiotic intervention [38]. Our results also indicated that INRs with CD4 count > 200 cells/μL had a higher CRP level yet not statistically significant. We proposed that the increased CRP level results from the excessive activated T cells [39]. Therefore, it is likely that there exists a positive correlation yet not significant between CRP and CD4^+^ T cell counts and CD4/CD8 ratio. Finally, We found that compared with subjects with CD4 count < 200 cells/μL, INRs with CD4 count > 200 cells/μL had less *Enterobacteriaceae* or *Fusobacteriaceae*. It is reasonable that *Enterobacteriaceae* and *Fusobacteriaceae* were positively related to TNF-α in HIV-infected INRs. However, in correlation analysis, TNF-α seems to be negatively correlated with *Enterobacteriaceae* and *Fusobacteriaceae*. Therefore, we speculate that there exists a false correlation between TNF-α and *Enterobacteriaceae* or *Fusobacteriaceae* in our study.

## Conclusions

Our results indicate that compared to INRs with CD4 count > 200 cells/μL, patients with CD4 count < 200 cells/μL had a lower CD4/CD8 ratio, lower nutritional status, and higher levels of pro-inflammatory cytokines including TNF-α, IP-10, and IL-1α. Alpha diversity of gut microbiota differed significantly between the two groups, and the relative abundance of *Ruminococcaceae* was markedly reduced in patients with lower CD4^+^ T cell counts. The correlation analysis indicated that CD4^+^ T cell counts were negatively associated with serum TNF-α and IL-1α levels and positively correlated with the relative abundance of *Ruminococcaceae*. Our findings provide a rationale and guidance for the subsequent nutritional intervention targeting gut microbiota to promote immune reconstitution in HIV-infected INRs.

## Supplementary Information

**Additional file 1.** Fig. S1. Beta diversity of gut microbiota in INRs. (A, B) Ordination of unweighted UniFrac distance between samples by PCoA (A) or NMDS (B). R andP values were derived from ANOSIM analyses of distance metrics. Fig. S2. Rectal microbial community composition of study participants. (A–C) A slight decrease in Bacteroidaceae (*P* = 0.3223) (A) and increase in Prevotellaceae (*P* = 0.7339) (B) and Prevotellaceae/Bacteroidaceae ratio (*P* = 0.4023) (C) were observed in the CD4 count < 200 cells/μL group compared to the CD4 count > 200 cells/μL group. The differences were statistically significant at a cut-off value of* P* < 0.05 (Mann-Whitney test).

## Data Availability

The dataset used in the manuscript is available from the corresponding author on reasonable request.
