# Supplementary material for: Association between CD4+ T cell counts and gut microbiota and serum cytokines levels in HIV-infected immunological non-responders
Source: BMC Infect Dis. 2021 Aug 3;21:742. doi: 10.1186/s12879-021-06491-z (PMC8336095; doi:10.1186/s12879-021-06491-z)
Supplement: Supplementary file 1 — Additional file 1. Fig. S1. Beta diversity of gut microbiota in INRs. (A, B) Ordination of unweighted UniFrac distance between samples by PCoA (A) or NMDS (B). R andP values were derived from ANOSIM analyses of distance metrics. Fig. S2. Rectal microbial community composition of study participants. (A–C) A slight decrease in Bacteroidaceae (P = 0.3223) (A) and increase in Prevotellaceae (P = 0.7339) (B) and Prevotellaceae/Bacteroidaceae ratio (P = 0.4023) (C) were observed in the CD4 count < 200 cells/μL group compared to the CD4 count > 200 cells/μL group. The differences were statistically significant at a cut-off value of P < 0.05 (Mann-Whitney test). [file 12879_2021_6491_MOESM1_ESM.doc]

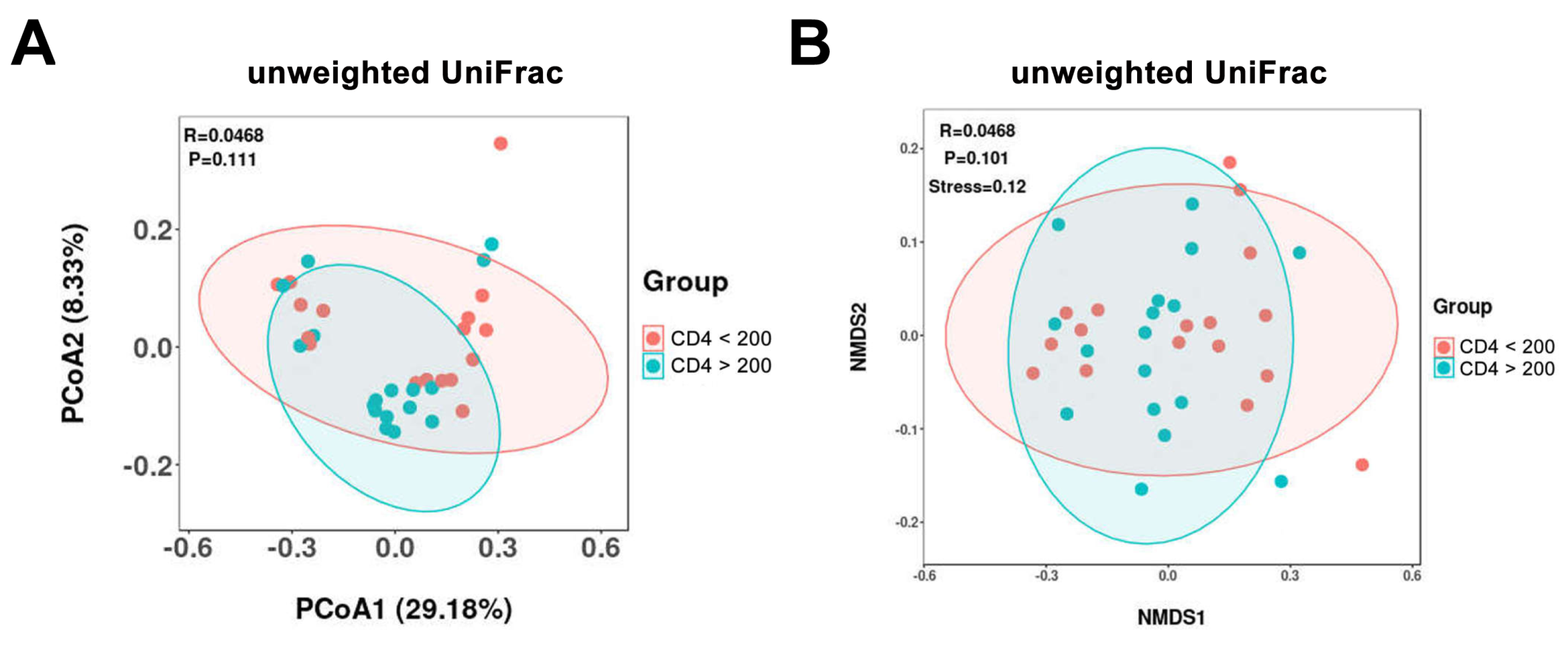


**Fig. S1. Beta diversity of gut microbiota in INRs.** (A, B) Ordination of unweighted UniFrac distance between samples by PCoA (A) or NMDS (B). R and *P* values were derived from ANOSIM analyses of distance metrics.


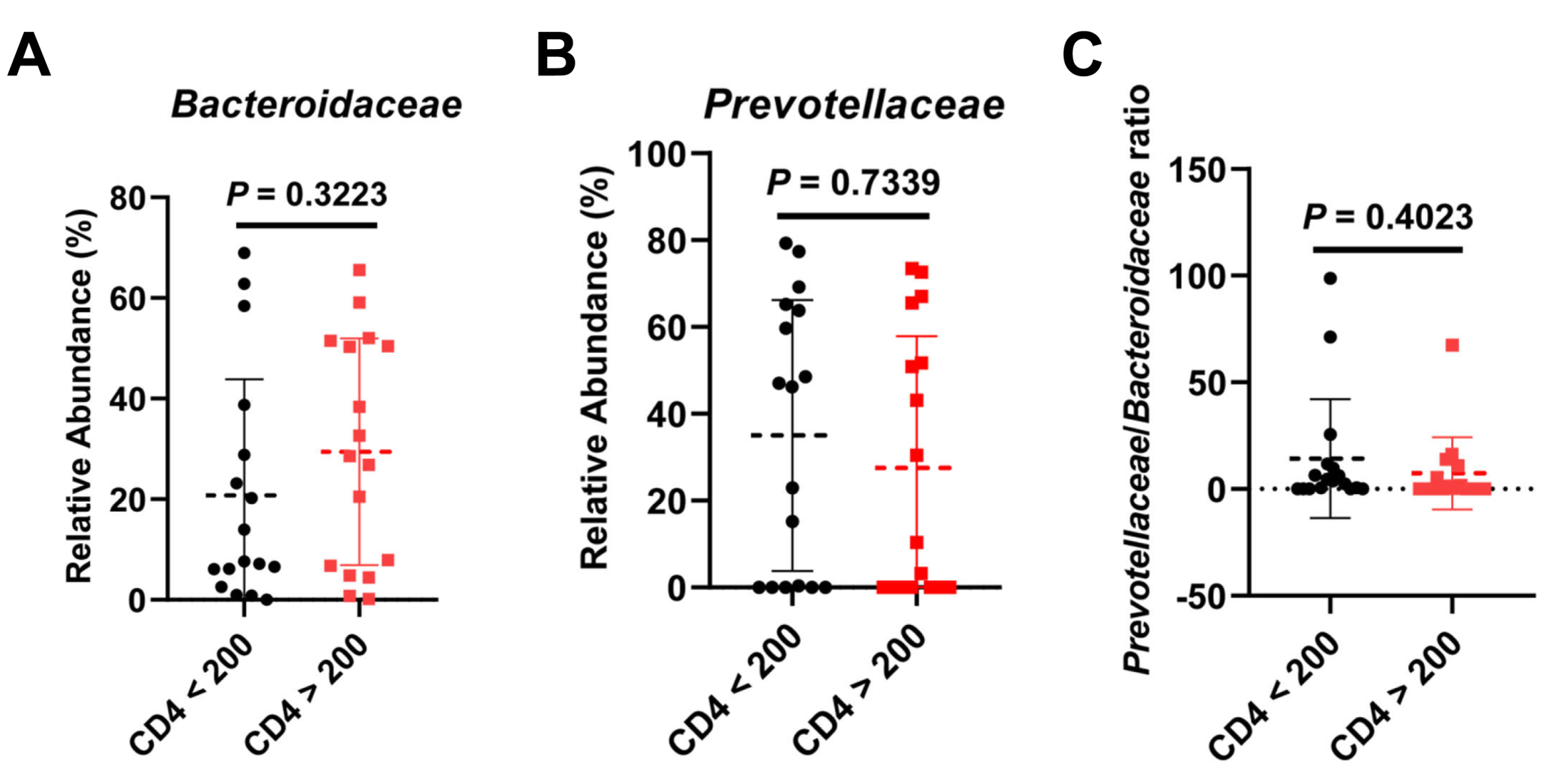


**Fig. S2. Rectal microbial community composition of study participants.** (A-C) A slight decrease in *Bacteroidaceae* (*P* = 0.3223) (A) and increase in *Prevotellaceae* (*P* = 0.7339) (B) and *Prevotellaceae*/*Bacteroidaceae* ratio (*P* = 0.4023) (C) were observed in the CD4 count < 200 cells/µL group compared to the CD4 count > 200 cells/µL group. None of the differences were statistically significant at a cut-off value of *P* < 0.05 (Mann-Whitney test).
